# The Human GP130 Cytokine Receptor and Its Expression—an Atlas and Functional Taxonomy of Genetic Variants

**DOI:** 10.1007/s10875-023-01603-7

**Published:** 2023-12-22

**Authors:** Yin-Huai Chen, Sarah van Zon, Alex Adams, Dirk Schmidt-Arras, Arian D. J. Laurence, Holm H. Uhlig

**Affiliations:** 1 Translational Gastroenterology Unit, University of Oxford, Oxford, UK; 2 Department of Biosciences and Medical Biology, University of Salzburg, Salzburg, Austria; 3 Biomedical Research Centre, University of Oxford, Oxford, UK; 4 Department of Paediatrics, University of Oxford, Oxford, UK

**Keywords:** *IL6ST*, GP130, STAT3, Gain-of-function, Loss-of-function

## Abstract

Genetic variants in *IL6ST* encoding the shared cytokine receptor for the IL-6 cytokine family GP130 have been associated with a diverse number of clinical phenotypes and disorders. We provide a molecular classification for 59 reported rare *IL6ST* pathogenic or likely pathogenic variants and additional polymorphisms. Based on loss- or gain-of-function, cytokine selectivity, mono- and biallelic associations, and variable cellular mosaicism, we grade six classes of *IL6ST* variants and explore the potential for additional variants. We classify variants according to the American College of Medical Genetics and Genomics criteria. Loss-of-function variants with (i) biallelic complete loss of GP130 function that presents with extended Stüve-Wiedemann Syndrome; (ii) autosomal recessive hyper-IgE syndrome (HIES) caused by biallelic; and (iii) autosomal dominant HIES caused by monoallelic *IL6ST* variants both causing selective IL-6 and IL-11 cytokine loss-of-function defects; (iv) a biallelic cytokine-specific variant that exclusively impairs IL-11 signaling, associated with craniosynostosis and tooth abnormalities; (v) somatic monoallelic mosaic constitutively active gain-of-function variants in hepatocytes that present with inflammatory hepatocellular adenoma; and (vi) mosaic constitutively active gain-of-function variants in hematopoietic and non-hematopoietic cells that are associated with an immune dysregulation syndrome. In addition to Mendelian *IL6ST* coding variants, there are common non-coding cis-acting variants that modify gene expression, which are associated with an increased risk of complex immune-mediated disorders and trans-acting variants that affect GP130 protein function. Our taxonomy highlights *IL6ST* as a gene with particularly strong functional and phenotypic diversity due to the combinatorial biology of the IL-6 cytokine family and predicts additional genotype-phenotype associations.

**Supplementary Information:**

The online version contains supplementary material available at 10.1007/s10875-023-01603-7.

## Introduction

The diagnostic and predictive power of clinical genetics depends on the evidence for solid genotype-phenotype associations and functional understanding of the consequences of genetic variants. Common genetic variation contributes to polygenic disease susceptibility via protein-coding variants (that modify protein function or stability) or more often intra- or intergenic variants in regulatory regions that modify mRNA expression (expression or protein quantitative loci in polygenic diseases) [1].

Advances in molecular biology and clinical genetics have led to the characterization of approximately 7000 Mendelian disorders that can be explained by protein-coding gain- or loss-of-function variants [2]. For each individual gene, there is typically a limited spectrum of Mendelian disease phenotypes even if there are multiple pathogenic mutations. This phenotypic spectrum is often determined by whether a given mutation confers a gain-of-function or loss-of-function on the translated protein giving rise to two potentially distinct clinical syndromes per gene (even if there is a spectrum of expressivity and incomplete penetrance). Furthermore, the widespread use of genomic sequencing has identified an increasing list of rare mutations that either have questionable clinical significance or are seemingly linked to an inconsistent variety of clinical phenotypes that are not obviously related.

In the immune system, combinatorial biology is a key principle that resulted in the evolution of families of cytokines with shared receptors [3]. Examples are the interleukin (IL)-6, IL-2, and IL-10 cytokine families that evolved around the GP130 (IL-6 family), the common gamma chain (IL-2 family), and the IL-10RB receptor (IL-10 family), respectively. Genetic variation in those cytokine receptor families poses experiments of nature since variation in the cytokine-specific components allows evolutionary adaptation of selective immune- and non-immune responses, whereas genetic variation in the common receptor components affects a potentially broader spectrum of cytokines. This is both a clinical challenge and an opportunity to understand the complexity of the underlying combinatorial biology [4].

GP130, encoded by *IL6ST*, is a shared signal transducing receptor for the IL-6 cytokine family [5, 6]. This cytokine family is both large and functionally diverse: members include IL-6 [7] and IL-27/IL-30 that regulate immune responses [8], IL-11 that regulates cells of mesenchymal origin and bone development [9], oncostatin M (OSM) that has roles in the immune system and hematopoiesis; leukemia inhibitory factor (LIF), cardiotrophin-1, cardiotrophin-like cytokine and ciliary neurotrophic factor, all of which regulate organ development [10, 11]. The number and functional diversity of the cytokines that signal via GP130 accounts for its pleiotropism. GP130 is ubiquitously expressed in all organs and virtually all cell types [12]. GP130 is central to multiple cellular processes including immune functions, hematopoiesis, organ development including lung and kidney, bone homeostasis, neural development, autonomous regulation, and cancer susceptibility and progression. The essential role of the IL-6 family of cytokines is reflected by the fact that *Il6st*-deficient mice die in utero at embryonic day 12.5 due to myocardial and hematopoietic defects [13] and essential biallelic loss-of-function variants in humans similarly cause intrauterine or postnatal lethal outcome [14].

Binding of IL-6 cytokines to the IL-6 receptor and the common receptor unit GP130 allows receptor complex assembly which ultimately results in the activation of signal transducer and activator of transcription 3 (STAT3) and in some instances STAT1 [7].

Structurally, GP130 has six extracellular domains: a N-terminal Ig-like domain (D1), two cytokine binding domains (D2-3), three membrane-proximal fibronectin type III domains (D4-6); a single transmembrane domain; followed by an intracellular cytoplasmic domain that contains multiple important functional sites including box1/2 motifs which are essential for the binding of Janus Kinases (JAKs), an internalization motif that is key for recycling GP130 from the cell surface, and four STAT3 binding sites. D2 and D3 are required for cytokine engagement by GP130, whereas D4 to D6 are necessary for a cytokine-dependent conformational change in the receptor that allows for association of the receptor subunits, ensuring proximity of the intracellular cytoplasmic domains to allow JAK cross-phosphorylation [15, 16]. Cytokines have a low binding affinity to GP130 alone and need prior binding to a cognate cytokine co-receptor. GP130 can either form hexameric or heterotrimeric complexes depending on the cytokine-co-receptor assembly. GP130 bound with IL-6 and IL-6Rα or IL-11 and IL-11Rα leads to a hexameric complex consisting of 2 copies of each component [17, 18]. IL-6Rα and IL-11Rα signaling is strictly dependent on GP130 for STAT activation. By contrast, GP130 bound with other IL-6 cytokine family receptors, including the signaling-competent OSM receptor (OSMR), LIF receptor (LIFR) form complexes consisting of a receptor heterodimer containing a single GP130 with their respective cytokines [19–21]. In most cases, this is a trimeric complex with a single cytokine molecule. This is except for IL-27, which is a heterodimer of IL-27p28 and Epstein-Barr Virus-induced 3 (Ebi3), and thus forms a heterotetramer with GP130 and the signaling competent IL-27Rα [21, 22].

**Fig. 1:**
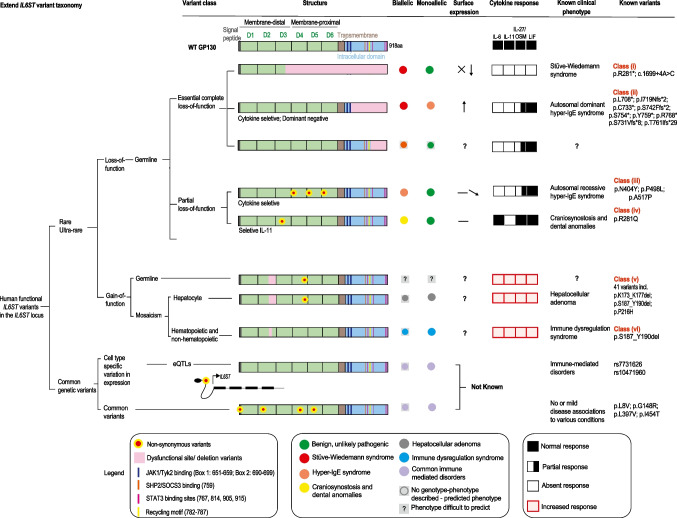
Extended *IL6ST* variant taxonomy and the current genotype-phenotype associations. Six classes of reported pathogenic *IL6ST* variants showing their mutant structures, surface expression, GP130-dependent cytokine signaling defects and clinical phenotypes. Novel variants can be potentially predicted based on the established classes

Here, we conduct a comprehensive investigation of *IL6ST* common variants and the currently known disease-causing mutations. Mutations due to loss-of-function of *IL6ST* variants can lead to a spectrum of distinct clinical phenotypes associated with different GP130-dependent cytokine signaling defects [5]. In recent years, several genotype-phenotype associations with loss-of-function [14, 23–26] or gain-of-function in GP130 have been identified [27–31]. As a consequence of the multiple and variable phenotypes and genotypes, a confusing taxonomy, terminology, and nomenclature has developed. Here, we assess the spectrum of genotype-phenotype associations and propose a unified taxonomy and nomenclature characterizing six classes of *IL6ST* variants with protein coding changes and distinct, well characterized functional outcomes (summarized in Fig. 1 and Table 1). We discuss that for some *IL6ST* variants (present in population databases or hypothetical) our knowledge on combinatorial biology allows prediction of phenotypes.

**Table 1:** Functional taxonomy of *IL6ST* variants in human

Pathogenic class/effect	Inheritance	Clinical phenotype	Number of patients	Number of variants	Variants Amino acid change	Variants Nucleotide change	Ref
Complete loss-of-function—impacts all IL6 family cytokines (class i)	Autosomal recessive	Extend Stüve-Wiedemann syndrome: severe bowing of long bones, respiratory dysfunction, dysautonomia, renal malformation pre- and peri-natal death except one	5–7^#^	2	p.R281* exon 13 skipping	c.841C>T c.1699+4A>G	[14]
Partial loss-of-function—IL6 and IL-11 cytokine dominant due to impact on hexameric cytokine-receptor complexes (class ii)	Autosomal recessive	Hyper-IgE syndrome: high IgE, eosinophilia, recurrent lung infections, pneumonia, defective acute-phase response, craniosynostosis, retained teeth, mild motor delays	3	4^##^	p.N404Y p.P498L p.A517P (p.Gly484_Pro518delinsArg)^####^	c.1210A>T c.1493C>T c.1549G>C (c.1552+3A>C)^####^	[24–26]
Partial loss-of-function—dominant negative impact on IL-6 and IL-11 cytokine due to impact on hexameric cytokine-receptor complexes (class iii)	Autosomal dominant	Hyper-IgE syndrome: high IgE, eosinophilia, recurrent lung infections, pneumonia, bronchiectasis, pneumatocele, skin abscesses, retained teeth, scoliosis	20	9	p.L708* p.I719fs p.C733* p.S742fs p.S754* p.Y759* p.T761fs p.Ser731Valfs*8 p.ARG768*	c.2121delT c.2155dup c.2199C>A c.2224dup c.2261C>A c.2277T>G c.2277_2281dup c.2190dup c.2303A>T	[23, 32]
Selective IL-11 loss-of-function (class iv)	Autosomal recessive	Craniosynostosis and dental anomalies: abnormal head shape, retained teeth requiring extraction of 14 teeth	1 (2^###^)	1	p.R281Q	c.842G>A	[33]
C-terminal loss-of-function variants (VUS)	N/A	Population databases (monoallelic)	2-3^#^	4	p.S789Ter, p.E899Ter, p.L906HfsTer28, and p.G913RfsTer1		-
Gain-of-function mosaicism with hepatocyte variants - Constitutively active (class v)	De novo	Inflammatory hepatocellular adenoma: benign tumor, increased CRP/SAA	258	41	Table 2-3	Table 2-3	[27–30]
Gain-of-function mosaicism with hematopoietic and non-hematopoietic (class vi)	De novo; mosaic	Neonatal onset immunodeficiency with autoinflammation and dysmorphy	1	1	p.S187_Y190del	c.557_571del	[31]
Expression eQTL	-	Associated with multiple diseases*	- (1365 homozygous)	1	G>A G>C	rs7731626 (*ANKRD55*, intron)	DICE; gnomAD
Common variants	Autosomal recessive or autosomal dominant	Not or associated with multiple conditions** Not reported in ClinVar	46; 3937; 2977; 1107 homozygous respectively	4	p.L8V p.G148R p.L397V p.I454T	c.C22G c.G442C c.C1189G c.T1361C	dbSNP; gnomAD

Six classes of pathogenic genotype-phenotypes of *IL6ST* variants are graded as class i–vi). ^#^ indicates confirmed genotypes and clearly affected patients by clinical phenotype but without confirmed genotypes; ^##^compound heterozygous; ^###^incomplete penetrance; ^#####^compound variant with complete loss-of-function effect, *thyroid diseases, autoimmune diseases, psoriasis, systemic lupus erythematosus, juvenile arthritis, rheumatoid arthritis, ulcerative colitis, Crohn’s disease, type I diabetes mellitus, common variable immunodeficiency, ankylosing spondylitis, celiac disease [34]. **Based on the current literature search, L8V has no SNP association; G148R is associated with traits of metabolic syndrome including fasting glucose, triglycerides, and systolic blood pressure (Italian population) [35]; L397V is associated with coronary artery disease (Russian), [36] multiple sclerosis (European) [37]; prostate cancer (African American) [38]; atherosclerosis [39]; I454T is associated with susceptibility to corneal infiltrative events of soft contact lens wearers with higher IL-6 concentration [40], and measles vaccine induced immunity in African Americans, but not in Caucasians [41]. VUS, variant of unknown significance

### Loss-of-Function Variants

There are several classes of loss-of-function *IL6ST* variants described in patients. The phenotypes can be grouped into four classes with three distinct clinical syndromes.

#### Class i) Biallelic *IL6ST* Variants with Complete Loss-of-Function Cause Extended Stüve-Wiedemann Syndrome

Biallelic essential loss-of-function *IL6ST* variants causing a complete lack of all GP130-dependent cytokine signaling including LIF signaling represent the most extreme phenotype. The lethal phenotype of *Il6st* knockout mice suggested a strong impact of *IL6ST* on normal embryonic development [13]. Consistent with this, the constraint metric from the Exome Aggregation Consortium/The Genome Aggregation Database (GnomAD) dataset indicates strong intolerance to loss-of-function variation for *IL6ST* (pLI: 0.998, highest is 1) [42].

Despite this prediction, complete loss of GP130 has been described in patients with pathognomonic features of Stüve-Wiedemann Syndrome, a lethal neonatal condition characterized by severe skeletal dysplasia and dysautonomia originally associated with loss-of-function mutations in LIFR [14, 43]. In exceptional instances, patients can survive early childhood [14]. These variants affect the extracellular domains of GP130 likely leading to the expression of truncated soluble GP130 variants (Fig. 1). Similarities of patients with loss of LIFR signaling and patients that had complete absence of GP130 suggest that LIF is essential during embryonic development. This is in accordance with a role of LIF/LIFR signaling at multiple steps of embryonic implantation and survival of embryonic stem cells in mice [44–46].

The healthy parents of patients with complete loss-of GP130 were found to be heterozygous for the pathogenic *IL6ST* variants. Cells of those parents show proportionally reduced surface levels of GP130 compared with healthy controls, suggesting that haploinsufficiency per se is not pathogenic [14]. In line with this, complete loss-of-function variants that affect the extracellular domains of GP130 can be found as an extremely rare heterozygous event in public databases such as GnomAD (p.V66GfsTer16, p.L27Ter, p.E316Ter, p.T555IfsTer3).

In addition to the loss-of-function stop-codon and frameshift variants, exon-skipping splice variants have been observed with complete loss-of-function in vitro [14]. In a heterozygous setting, these variants do not cause a phenotype, but in homozygous setting such variants are pathogenic and cause a Stüve-Wiedemann syndrome phenotype (intronic splice site variant within the *IL6ST* locus, c.1699+4A>G). Loss of exon 13 in the c.1699+4A>G splice variant abrogates folding of GP130 membrane-proximal domain D6 impairing transmembrane signal transmission [14].

##### ACMG Classification

Due to the strong evidence based on at least five confirmed (potentially 8) individuals of 3 independent families with biallelic variants, two biallelic genetic variants in patients and well-established functional studies, we grade these biallelic complete loss-of-function as strong pathogenic variants. Phenotypes of patients with monoallelic and biallelic *IL6ST* variants in the extracellular domain (as well as those that would delete the membrane domain), can likely be predicted.

#### Class ii) Biallelic Loss-of-Function *IL6ST* Variants That Largely Affect IL-6 and IL-11 Signaling Cause Autosomal Recessive Hyper-IgE Syndrome

Three patients were described with biallelic missense variants in *IL6ST* (p.N404Y, p.P498L, p.A517P) displaying symptoms that resemble HIES (defined as HIES4; MIM#618523). Two patients carried homozygous variants (p.N404Y, p.P498L) and a third a compound heterozygous variant with a non-synonymous variant p.A517P and a complete loss-of-function variant c.1552+3A>C splice variant leading to exon 12 skipping (Fig. 1) [24–26]. All patients were phenotypically similar to the classical autosomal dominant form of HIES associated with heterozygous mutations of *STAT3*, characterized by craniosynostosis and dental anomalies together with recurrent bacterial infections. The patients’ phenotype could be explained largely by a loss of IL-6, and IL-11 signaling with potential contributions of the partial defect seen in the IL-27 response, suggesting that absence of these cytokines plays a key role in the phenotype of STAT3-associated HIES. This is consistent with the literature: IL-6 and STAT3 activation are linked with T cell expression of IL-17 (Th17 cells), which are key for protection against fungal and extracellular bacterial infections [10]. IL-6Rα deficiency has also been associated with enhanced T cell expression of IL-4, 5, and 13 (Th2 cells) associated with atopic dermatitis and elevated IgE expression [47]. Loss of IL-27 expression may also enhance Th2 cell development [48]. IL-11 plays a role in bone development accounting for the cranial abnormalities seen in STAT3-associated autosomal dominant HIES [9].

This phenotype contrasts with that seen with the class (i) complete loss of GP130 mutations. This is reflected in a degree of cytokine selectivity associated with these mutations since there is remaining signaling downstream of LIF and to a lesser extent OSM and IL-27 compared with the defect in IL-6 and IL-11 signaling [26]. The three non-synonymous missense variants disrupt the extracellular membrane domain interfaces of GP130, which is likely required for stabilization of the GP130-IL-6Rα-IL-6 and GP130-IL11Rα-IL-11 hexameric complexes but partially spare the tetrameric GP130-IL-27Rα-IL-27, and the trimeric GP130-LIFR-LIF and GP130-OSMR-OSM complexes, preserving LIF, OSM, and IL-27 signaling [26].

##### ACMG Classification

Despite well-established functional results and the plausible mechanism, the low patient number with single patient per variant suggest moderate evidence: likely pathogenic. Novel variants cannot be predicted at the moment and need to be validated. Monoallelic variants are not pathogenic.

#### Class iii) Monoallelic Loss-of-Function *IL6ST* Variants That Affect IL-6 and IL-11 Signaling Cause Autosomal Dominant Hyper-IgE Syndrome

Certain heterozygous mutations of *IL6ST* cause an autosomal dominant form of HIES that is clinically similar but genetically distinct from the classical or type I form of autosomal dominant *STAT3* HIES. Twelve patients with HIES carrying heterozygous dominant negative *IL6ST* mutations have been reported [23]. A recent publication described further 2 variants in 8 patients [32]. These mutations are stop-codon or frameshift mutations (leading to premature stop codons) in the intracellular cytoplasmic domain (Fig. 1). Those mutations retain the GP130 transmembrane domain, and box 1/2 motifs but typically lack all four STAT3 binding sites and the recycling/internalization motif [23]. The recently identified p.R768* variant lacks the three most distal STAT3-binding residues [32]. As a result of the absent internalization domain and the defective STAT3 binding sites, the p.R768* variant accumulates on the cell surface and cause defective STAT signaling due to stochiometric enrichment of the LOF variants. These variants cause a net loss-of-function effect that dominantly affects cellular responses to IL-6 and IL-11 while OSM and LIF signaling is relatively spared with a minor loss of signaling. This sparing can be explained by the stoichiometry of one single non-functional copy of GP130 having a stronger effect on the hexameric receptor complexes (that require two GP130) compared to the tri- and tetrameric GP130 complexes (that require one GP130 chain) [49, 50]. It is uncertain whether this minor loss of OSM/LIF signaling contributes to the phenotype of this patient group. Finally, there is functional variation among the variants that lack the internalization domain since the variant, p.S731Vfs*8 does not accumulate at a similar level at the cell surface compared to other variants in this domain and is associated with a milder phenotype [32].

No individual with a heterozygous loss-of-function variant that abrogates the recycling motif and the STAT3 binding sites has been described in any population-based database.

##### ACMG Classification

Due to the strong evidence based on 20 individuals, 9 variants and well-established functional studies, we grade these complete loss-of-function as strong pathogenic variants. Future variants can likely be predicted.

#### Class iv) Biallelic Partial *IL6ST* Loss-of-Function That Selectively Affects IL-11 Signaling Cause Autosomal Recessive Craniosynostosis and Dental Anomalies

A patient with craniosynostosis and dental anomalies but no immune dysregulation carried a homozygous missense variant p.R281Q [33] (Fig. 1). Primary fibroblasts were not available, but this variant exclusively affected IL-11 signaling in patient-derived lymphocytes transduced with *IL11RA* as well as transfected HEK293T cells in a heterologous assay system. The variant is not fully penetrant since the homozygous mother had no clinical defect. A second patient with homozygous *IL6ST* p.R281Q with pan-craniosynostosis was recently identified (Mcmanus & Bhoj, personal communication). A mouse knock-in with the 281 homologous variant p.R279Q caused a phenotype of mildly deranged cranial development like that seen in *Il11ra*-deficient mice.

No individual with a homozygous variant p.R281Q variant has been described in any population-based database.

##### ACMG Classification

Due to the replicated phenotype and clear functional defects based on patient-derived and heterologous models as well as mouse data, we classify this variant as likely pathogenic with incomplete expressivity/penetrance.

#### Loss-of-Function Variants in the C-Terminal Tail of GP130

Individuals with heterozygous essential loss-of-function variants that maintain the recycling motif and at least one of the four STAT3-binding residues at tyrosine positions can be found in population databases such as GnomAD (p.S789Ter, p.E899Ter, p.L906HfsTer28, p.G913RfsTer10). Those variants can have some effect on IL-6 and IL-11 signaling in vitro [23], but genotype-phenotype associations are lacking for heterozygous individuals.

##### ACMG Classification

Monoallelic variants that affect the C-terminal tail are variants of unknown significance since genotype-phenotype associations are lacking. Once phenotype data on individuals with mono- or biallelic C-terminal tail mutations are described, and primary cells are tested, those variants can be grouped into the classes (i), (ii), and (iii) or classified as non-pathogenic.

#### GP130 Isoforms

There are several physiological isoforms of GP130 that exist in different tissues alongside the canonical wild-type GP130 (Uniprot database). Many of these are predicted loss-of-function while the physiological role of these isoform variants is not clear.

### Gain-of-Function Variants

Constitutively active GP130 germ line variants have not been reported. However, there are two classes of tissue restricted gain-of-function *IL6ST* variants described in patients, either in hepatocytes, or hematopoietic cells. Somatic mosaicism is defined as postzygotic mutation that may occur at any developmental stage or in adult tissues [51].

#### Class v) Gain-of-Function Variants—Somatic Mosaicism in Hepatocytes

**Fig. 2:**
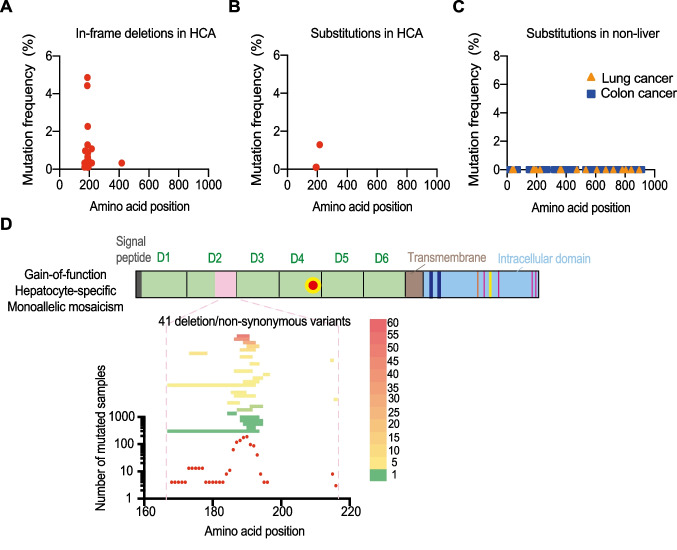
Identification of putative constitutively active *IL6ST* gain-of-function variants in liver vs non-liver cancers. **A**, **B** Frequency of *IL6ST* mutation due to in-frame deletions or non-synonymous substitutions in HCA. Meta-analysis is based on four HCA cohorts and COSMIC database. **C** Frequency of *IL6ST* mutation due to non-synonymous substitutions in colon and lung cancer. Meta-analysis is based on COSMIC, TCGA, and ICGC databases. **D** Deletion length showing majority of the variants cluster in the region of p.T183 to p.E195 in D2. D, Domain. Color-coded heat map corresponds to the number of mutated samples at the specified deleted site

Constitutively active *IL6ST* somatic gain-of-function variants have been described in inflammatory hepatocellular adenoma (IHCA) [27–30]. IHCA is a subgroup of hepatocellular adenoma (HCA), which are rare benign tumors that develop in a normal liver, typically in young female patients following contraceptives or exposure to estrogen [52]. To identify the spectrum of constitutively active *IL6ST* gain-of-function variants, we performed a meta-analysis of four independent cohorts that investigated hepatocellular adenoma [27–30] and COSMIC database entries that investigated liver tumors, specifically HCA. A total of 926 liver samples were counted, generating a list of 36 different small in-frame deletions (Table S1). The most frequent in-frame heterozygous somatic *IL6ST* deletion was p.S187_Y190del with a frequency of 4.86% (45/926) (Fig. 2A and Table S1). In addition, we recorded 5 different non-synonymous substitutions with p.P216H being the most common (Fig. 2B and Table S1).

Using cancer genomic databases (Catalogue of Somatic Mutations in Cancer (COSMIC), The Cancer Genome Atlas Program (TCGA) and International Cancer Genome Consortium (ICGC)), we confirmed that the *IL6ST* mutations identified from the meta-analysis do not occur in either lung or colon tumors (Fig. 2C) suggesting they are specific to HCA. This is consistent with the investigation of other lung cancer cohorts in which *IL6ST* mutations are not common genetic variants [53, 54]. Overall, most of the variants (total 41 variants) cluster in the region of p.T183 to p.E195 and p.Y190 is the most commonly mutated deleted site (Fig. 2D). None of the 36 different small in-frame deletions and the p.P216H HCA-specific mutations in *IL6ST* was present in public databases including GnomAD [55] and Bravo/TOPMed (https://topmed.nhlbi.nih.gov). Around 60% of IHCA have in-frame somatic deletions of *IL6ST*. These variants lead to STAT3 activation and upregulation of acute-phase proteins and suppressor of cytokine signaling (SOCS) 3 in a ligand-independent fashion [28–30]. GP130 dimerization on its own is not sufficient to trigger activation in the absence of cytokine binding [56] and unliganded GP130 has been proposed to exist as an inactive, preformed homodimer at the plasma membrane [57, 58]. However, p.Y186_Y190del and p.A418_F421del variants contributed to GP130 activation by showing their capacity to homo- or hetero-dimerize with another GP130, either as wild type (WT) or mutant, in the absence of IL-6 or OSM [29, 30]. There is evidence that gain-of-function mutations have in common that they compromise structure or conformational stability of the cytokine binding module and stable interaction of the two cytokine binding domains (D2, D3), which results in constitutive activity [56]. Selective hepatocyte-specific gain-of-function somatic mutations likely cause an inflammatory phenotype by activated acute-phase response. One of the functions of IL-6 is to regulate the induction of acute-phase proteins in hepatocytes. These proteins are part of an evolutionary conserved first-line defense to environmental challenges and include the acute-phase proteins C-reactive protein (CRP), serum amyloid A (SAA) and fibrinogen. Members of the SAA family are among the most highly inducible acute-phase proteins [59] and were shown to be elevated in chronic inflammatory diseases [60, 61]. SAA1/2 were suggested to exert antibacterial and immunoregulatory function. Transgenic mice bred to express a hepatocyte-specific constitutive active gp130 had increased expression of acute-phase proteins that led to a serum amyloid A amyloidosis with age. There was a stimulated recruitment of myeloid cells to the liver and Kupffer cells (hepatic myeloid cells) adopted an enhanced bactericidal phenotype that protected animals from bacterial challenge [62]. This study directly supports a hepatocyte gain-of-function phenomenon in HCA, where hepatocyte-specific GP130 drives an inflammatory phenotype. In the same line, SAA proteins can stimulate naive CD4+ T helper cells to adopt a pro-inflammatory pathological Th17 phenotype [63].

##### ACMG Classification

Due to the large number of IHCA patients and the tumor-specific effects, we classify deletions in the region p.T183 to p.E195 and the p.P216H variant as pathogenic. There is no evidence for germline inheritance. Novel deletion variants in the T183 to E195 region can likely be predicted.

#### Class vi) Gain-of-Function Somatic Mosaicism in Hematopoietic Cells

A child with heterozygous *de novo IL6ST* p.S187_Y190del variant was recently reported exhibiting neonatal onset immunodeficiency with autoinflammation and dysmorphia [31]. The p.S187_Y190del variant was previously identified as a constitutively active *IL6ST* variant in HCA cohorts [27–30]. In this patient, the mosaic variant likely arose early in embryonic development since it was estimated in approximately 15–40% of different cell types (no exact copy analysis in primary cells). Epstein-Barr virus transformed lymphoblastoid cell lines from the patient showed constitutive STAT3 activation which could be inhibited by the JAK inhibitors, ruxolitinib, and tofacitinib [31]. In contrast to murine studies where B-cell-specific expression of the constitutively active artificial Lgp130 variant induced mature lymphoma and plasmacytoma [64, 65], no signs of B-cell malignancies were reported for this patient. Interestingly, severe pulmonary (auto)inflammation was observed in mice with constitutive GP130 signaling in T-cells that was linked to enhanced Th17 formation [66]. It is therefore tempting to speculate that the autoinflammation observed in the patient is rather linked to GP130-mediated T-cell hyperactivity.

To search for additional patients that harbor putative constitutively active *IL6ST* gain-of-function variants as indicated by IHCA-associated variants, we screened genetic and phenotype-specific databases including patients with congenital early-onset inflammatory and non-inflammatory conditions and were not able to identify additional germline heterozygous or mosaic variant that affects peripheral blood cells. However, infants with a low percentage mosaicism might initially be less affected (and the mosaicism might be undetected due to filtering and quality control in the variant calling algorithms) but develop a more severe phenotype later in life as immune cells with the *IL6ST* mutation accumulate through cellular expansion over time.

##### ACMG Classification

Although there is only a single patient with the p.S187_Y190del variant, the corresponding evidence of the genetic variant in IHCA and the absence of any of the gain-of-function variants in population-based datasets suggests that the variant is likely pathogenic. Novel deletion variants in the T183 to E195 region variants and the p.P216H variant can be predicted to have a similar pathogenic effect based on the high confidence in the spectrum of IHCA-associated variants.

### Common Non-coding eQTLs That Affect *IL6ST* Transcription

**Fig. 3:**
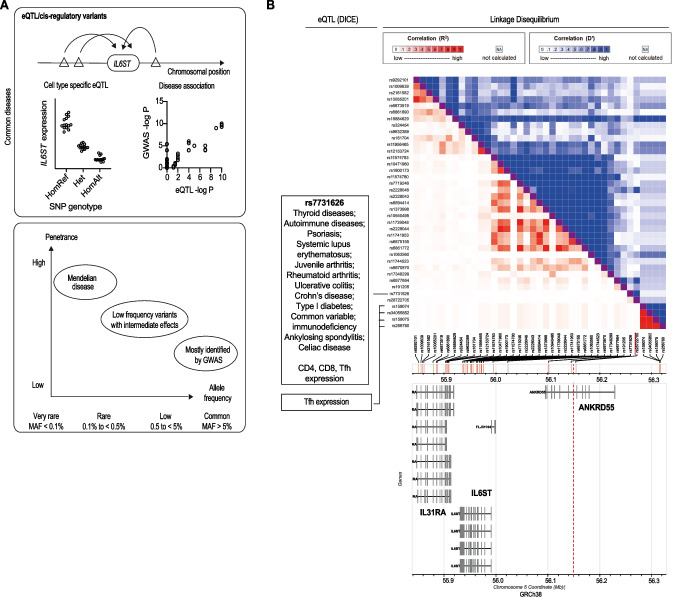
Analysis of *IL6ST* eQTL and common variants. **A** *IL6ST* eQTL, common variants, and isoforms act to alter gene expression. **B** Main: Heatmap showing correlation between variants in the *IL6ST* locus with correlations measured by *r*^2^ (red) and D′ (blue) demonstrating linkage disequilibrium—where the associations between variants are non-random. Left: known cell/tissue type expression and disease association (data derived from the ClinVar, Decipher, OMIM, and ClinGen disease databases). Below: Map showing position of variants aligned to transcripts of *IL31RA*, *IL6ST*, and *ANKRD55*

In contrast to monogenic disorders that are inherently rare, disease susceptibility may be driven by common protein-coding variants with small impact on GP130 function or non-coding variants in the *IL6ST* locus (or elsewhere) that affect *IL6ST* expression (Fig. 3A). *IL6ST* is differentially expressed across cell types, where fibroblasts show the highest expression of *IL6ST* (GTEx Portal). Common variants in the locus of *IL6ST* act as expression quantitative trait loci (eQTL) which alter gene expression of *IL6ST* in specific cell types (Fig. 3A). Allele frequencies of those variants are population specific; for instance, minor allele frequency of *IL6ST* SNP eQTL rs7731626 (5-55444683-G-A) ranges from 0.36 in non-Finnish European populations, to 0.19 in African/African-American, and 0.08 in east Asian populations (GnomAD).

The intronic variant rs7731626 (5-55444683-G-A), located in the wider *IL6ST* and ANKRD55 locus (Fig. 3B), is associated with immune-mediated diseases such as Crohn’s disease, rheumatoid arthritis [67], multiple sclerosis [68], systemic lupus erythematosus, and common variable immunodeficiency as suggested by Open Targets Genetics [69] and Database of immune cell eQTLs expression epigenomics (DICE) [34]. The protective allele rs7731626 (A) is associated with decreased expression of *IL6ST* in CD4+ T-cells (Beta = − 0.79, FDR = 5.0 × 10^−9^) [70]. It is not clear how the transcription differences relate to protein expression. The *IL6ST* eQTL is further supported by Promoter Capture Hi-C data that shows a significant chromatin interaction between the variant, located within an intron of ANKRD55, and the promoter of *IL6ST* specifically in naive CD4+ T-cells [70, 71]. The CEDAR dataset that included 9 cell types (lymphoid, myeloid, and intestinal cell types) and compared eQTL association pattern and disease association pattern reported that *IL6ST* and ANKRD55 polymorphisms from T cells are implicated in Crohn’s disease as a risk locus (ϑ = 0.9, *p* = 0.02; ϑ > 0.6 with low empirical values indicate strong correlations) [72]. A positive correlation between disease association pattern and eQTL association pattern was observed in CD4+ and CD8+ T cells suggesting that the variant controls *IL6ST* expression as a cis-acting eQTL. Again, it is not clear how the transcription differences relate to protein expression.

In addition, variants within the *IL6ST* locus are associated with atherosclerotic disease in a sub-phenotype of families with myocardial infarction: rs1900173 (5-55240006-A-T) has an odds ratio of 1.92 (95% CI 1.03–3.6) for ostium stenosis (*p* = 0.0023) [34]. Although it is unclear how these variants impact on *IL6ST *expression, mechanistic studies suggest that this is driven by a hepatocyte-dependent acute-phase response. Using hepatocyte-specific gp130 deficient mice on an atherosclerosis-prone background, the mice were protected from atherosclerosis due to reduced expression of acute-phase proteins and less macrophage recruitment to vessel walls [39].

These data together suggest that *IL6ST* eQTLs differentially regulate gene expression in a cell or tissue type specific manner. The context- and cell-type-specific expression might explain the association of the *IL6ST* locus with several immune-mediated disorders. However, experimental evidence is still lacking that elevated *IL6ST* mRNA expression levels translate to increased cell surface protein levels or impact on signaling.

## Limits of the Current Understanding of *IL6ST* Variation

As outlined above, our understanding of published genotype-function-phenotype associations and knowledge of the biochemical properties of those variants in vitro, allow to predict the impact of some additional coding variants in *IL6ST* within the same genotype-phenotype class. Additional phenotypic heterogeneity will likely arise due to somatic mosaicism. Like STAT3-associated HIES [73], *IL6ST* loss-of-function variant mosaicism is probably present in some patients with *IL6ST* autosomal dominant HIES. The allelic combination can contribute to further variation. While monoallelic essential loss-of-function variants that lack the recycling motif cause autosomal dominant HIES, biallelic variants would hypothetically almost certainly cause Stüve-Wiedemann syndrome. Loss-of-function variants in the C-terminal end of GP130 that maintain a functional recycling motif are unlikely to cause an autosomal dominant HIES while (yet undescribed) biallelic variants or compound heterozygous variants may cause aspects of Stüve-Wiedemann syndrome or HIES.

Parallel to the IL-11 cytokine-selective variant in GP130, it is likely that additional classes of *IL6ST* variants with a different cytokine selectivity exist. This is currently not possible to predict using structural modeling. It is currently not known whether germline non-mosaic de novo gain-of-function variants that affect hematopoietic and non-hematopoietic cells are lethal in utero and/or cause severe developmental and immune-dysregulation defects.

### Transacting Genetic Variants That Affect GP130 Protein Expression

Although not the focus of this review, it is important to note that rare and common trans-acting genetic variation distant from the *IL6ST* gene locus can affect GP130 function.

Patients with biallelic loss-of-function variants in phosphoglucomutase 3 (PGM3) present with a congenital disorder of glycosylation. This enzyme catalyzes the conversion of N-acetyl-glucosamine (GlcNAc)-6-phosphate into GlcNAc-1-phosphate required for several glycosylation responses. Among other proteins, loss of PGM3 activity affects glycosylation of GP130 and is associated with reduced GP130 surface expression and defective STAT3 phosphorylation after stimulation with IL-6, which accounts for the HIES-like phenotype seen in these patients [74]. PGM3 deficiency is associated with psychomotor retardation and variable blood cell abnormalities (includingeosinophilia). These additional features can be accounted for by the impairment of other members of the GP130 family, while the expression of other receptors is likely to be affected by a defect in glycosylation [75].

There is evidence for common trans-acting expression protein trait locus (ePTL) activity where rs579459 located in the ABO locus on chromosome 9q34.2 affects the soluble GP130 in serum with unknown consequences for GP130-dependent cytokine signaling [76]. It is currently not clear whether these serum protein concentration effects are mediated by impact on GP130 O-glycosylation, cellular damage, inflammation, or shedding of GP130 from the cell surface.

## Functional Analysis of GP130 Variants—a Guide to Assay Systems and Cell-Type-Specific Effects

In light of the phenotypic and functional variability of genetic variants in *IL6ST*, it is essential to perform functional validation in variants of unknown significance. The functional characterization of *IL6ST* variants should ideally include GP130 cell surface expression and cytokine stimulation experiments across multiple GP130 cytokines, whenever possible using primary cells (and complemented by heterologous systems when needed) [14, 23–26].

Measuring STAT3 phosphorylation by FACS and STAT3 luciferase reporter assays were the most common ways of assessing cytokine responses in primary cells of patients with *IL6ST* variants (including T cells, B cells, monocytes, fibroblasts) as well as cell lines (transfection of HEK293T or Hep3B cells). In transfection systems, the IL-6 family cytokine response can be analyzed by flow cytometry as well as by STAT3 luciferase reporter assay [23]. The overall comparability of those assays is robust, although there are noticeable quantitative differences in response to GP130-dependent cytokine stimulations assessed by pSTAT3 flow cytometry and STAT3 reporter luciferase assay [25]. Differences between those assays can occur due to different kinetics capturing a single time point by flow cytometry versus an integrated longitudinal effect of the transcriptional response in the reporter assay [14, 23–26].

## Therapeutic Implications of a Genetic Diagnosis

The therapeutic implication for patients with pathogenic *IL6ST* variants depends on the underlying condition ranging from intensive neonatal and supportive care in patients with Stuve-Wiedemann syndrome, antibiotic and antifungal treatment, and prophylaxis in patients with *IL6ST*-associated HIES, surgery in patients with craniosynostosis to immunomodulatory treatment in patients with gain-of-function variants.

The success of kinase inhibitors in the fields of oncology and immunology raises the possibility of targeted therapies for *IL6ST* gain-of-function conditions. Gain-of-function variants can potentially be treated using a variety of JAK1 or JAK1/2 inhibitors that are clinically available for the treatment of rheumatoid arthritis, myeloproliferative disease, and inflammatory bowel disease [77, 78] since GP130 relies on the recruitment of JAK1 and JAK2 for downstream signaling.

Dupilumab that targets IL-4 and IL-13 signaling has been used in a patient with autosomal dominant *IL6ST*-related HIES. This led to improved eczema and reduced IgE levels, but did not affect the patient’s asthma [32].

Signaling-modifying targeted treatments of patients with loss-of-function IL6ST variants are much more challenging to develop. Many of the loss of function groups are analogous to classical type 1 HIES caused by STAT3 variants. Allogeneic bone marrow transplantation has been used to treat classical type 1 HIES, but the correction is limited to the hematopoietic compartment and this therapy carries a significant mortality rate that makes the therapy hard to justify except in the most severe cases [79]. It is likely to have a similar role in patients with loss-of -function *IL6ST* variants that are limited to the hematopoietic system, but this will not be effective in treating the multi-organ manifestations of severe Stüve-Wiedemann syndrome.

The JAK2 agonist Eltrombopag is licensed for the treatment of immune thrombocytopenia [80]. Eltrombopag is a small molecule that binds the thrombopoietin receptor and enhances JAK2 and subsequent STAT5 signaling to promote platelet production [81]. As GP130 is in the same family of type I/II cytokine receptors as the thrombopoietin receptor, this raises the possibility that drugs with a similar mechanism may be used to enhance GP130-dependent JAK1/JAK2 signaling in patients with a partial loss-of-function *IL6ST* mutations.

There is currently no clinical relevance of common variants in the *IL6ST* locus, although it is noticeable that several of the disorders such as Crohn’s disease [82] and rheumatoid arthritis associated with the rs7731626 (5-56148856-G-A) variant affect *IL6ST* expression. It needs to be shown, whether those variants have an impact on IL-6 signaling within T cells and whether those patients might benefit particularly from IL6R targeting therapies [83] or GP130 targeting therapies [84].

## Conclusions

Our taxonomy has relevance for clinical genetics and the functional validation process for rare genetic variants in *IL6ST* with Mendelian inheritance or mosaicism. The discovery of somatic gain-of-function variants in hepatocellular tumors in 2008 [30] and loss-of-function monogenic *IL6ST* variants in 2017 [24] has been the beginning of a process that has been of prognostic value for a small but increasing number of patients and their families. Our taxonomy of functional *IL6ST* variants spanning monogenic disorders with distinct and extreme clinical phenotypes and common *IL6ST* variants associated with immune-mediated disorders informs on the differential contribution of GP130-dependent cytokines.

### Supplementary Information

ESM 1 (DOCX 47 kb)

## Abbreviations

ACMG: American College of Medical Genetics and Genomics
COSMIC: Catalogue of Somatic Mutations in Cancer
D: Domain
DICE: Database of Immune Cell Expression, Expression quantitative trait loci (eQTLs) and Epigenomics
ePTL: Expression probabilistic trait locus
eQTL: Expression quantitative trait locus
GnomAD: The Genome Aggregation Database
GTEx: Genotype-tissue expression
HCA: Hepatocellular adenoma
HIES: Hyper-IgE syndrome
ICGC: International Cancer Genome Consortium
IHCA: Inflammatory hepatocellular adenoma
IL: Interleukin
JAKs: Janus Kinases
LIF: Leukemia inhibitory factor
LIFR: Leukemia inhibitory factor receptor
OSM: Oncostatin M
OSMR: Oncostatin M receptor
PGM3: Phosphoglucomutase 3
STAT3: Signal transducer and activator of transcription 3
TCGA: The Cancer Genome Atlas Program
WT: Wild type
