# Supplementary material for: The Human GP130 Cytokine Receptor and Its Expression—an Atlas and Functional Taxonomy of Genetic Variants
Source: J Clin Immunol. 2023 Dec 22;44(1):30. doi: 10.1007/s10875-023-01603-7 (PMC10746620; doi:10.1007/s10875-023-01603-7)
Supplement: Supplementary file 1 — (DOCX 47 kb) [file 10875_2023_1603_MOESM1_ESM.docx]

**Supplementary Table 1. Putative constitutively active *IL6ST* variants due to in-frame deletion and non-synonymous substitution identified in HCA tumor samples.** *IL6ST* mutations due to in-frame deletions and non-synonymous substitutions from four independent cohorts ^1, 2, 3, 4^ and COSMIC were counted. Only HCA tumor samples were included in the meta-analysis. There are 36 different in-frame deletions and 5 non-synonymous substitutions, with the most frequent mutation shown from the top of the list. Some tumors harbor both *IL6ST* and *CTNNB1* mutation and those were counted ^1, 3^. Total number of analyzed HCA samples is 926**.** Frequency of mutation is calculated from the total number per mutation/total number of HCA cases.

| **Amino acid**  **change** | **Nucleotide**  **change (c.)** | **Rebouissou et al** | **Poussin et al** | **Calderaro et al** | **Pilati et al** | **COSMIC** | **Total** |
| --- | --- | --- | --- | --- | --- | --- | --- |
| **In-frame deletion** |  |  |  |  |  |  |  |
| p.S187_Y190del | 560_571del | 6 | 12 |  | 14 | 13 | 45 |
| p.Y186_Y190del | 557_571del | 4 | 9 | 4 | 9 | 15 | 41 |
| p.V189_V192del | 565_576del | 3 | 5 | 1 | 5 | 7 | 21 |
| p.T188_V192delinsIle | 563_574del | 2 | 4 |  | 2 | 4 | 12 |
| p.D215del | 643_645del | 1 | 3 |  | 3 | 3 | 10 |
| p.K173_D177del | 518_532del | 1 | 2 | 1 | 2 | 3 | 9 |
| p.Y190_N193del | 567_578del |  | 2 |  | 2 | 4 | 8 |
| p.T188_F191del | 562_573del |  | 2 |  | 2 | 2 | 6 |
| p.V189_V192del | 564_575del |  | 2 |  | 2 | 2 | 6 |
| p.Y190_N193del | 568_579del |  | 1 | 1 | 1 | 2 | 5 |
| p.E195_V196del | 583_588del | 1 | 1 |  | 1 | 1 | 4 |
| p.F191_N193del | 570_578del |  | 1 |  | 1 | 2 | 4 |
| p.V184_S187delinsAla | 551_559del | 1 | 1 |  | 1 | 1 | 4 |
| p.V189_N193delinsAsp | 566_577del |  | 1 |  | 1 | 2 | 4 |
| p.Y186_V192delinsPhe | 557_574del | 1 | 1 |  | 1 | 1 | 4 |
| p.V189_V192del | 564_576delinsA |  | 1 |  | 2 | 1 | 4 |
| p.A418_F421del | 1252_1263del |  |  |  | 2 | 1 | 3 |
| p.D185_V189del | 552_566del |  | 1 |  | 1 | 1 | 3 |
| p.K168_N193del | 503_580del |  | 1 |  | 1 | 1 | 3 |
| p.V189_N193del | 565_579del |  |  |  |  | 3 | 3 |
| p.T188_V192del | 563_577del |  | 1 |  | 1 | 1 | 3 |
| p.V192_I194del | 575_583del | 1 |  |  |  | 2 | 3 |
| p.P216del | 646_648del |  | 1 |  | 1 | 1 | 3 |
| p.F191_I194delinsLeu | 573_582delinsG |  | 1 |  | 1 |  | 2 |
| p.F191_N193del | 571_579del |  | 1 |  | 1 |  | 2 |
| p.S187_F191del | 559_573del | 1 |  |  |  | 1 | 2 |
| p.V189_I194delinsAspVal | 566_580delinsATG |  | 1 |  | 1 |  | 2 |
| p.V189_V192delinsPhe | 565_574delinsT |  | 1 |  | 1 |  | 2 |
| p.Y186_F191delinsIle | 556_571delinsA |  | 1 |  | 1 |  | 2 |
| p.Y190_N193delinsSer | 569_578delinsC |  | 1 |  | 1 |  | 2 |
| p.N193del | 557_579del | 1 |  |  |  |  | 1 |
| p.T188_F191del, V192T | 564_575del | 1 |  |  |  |  | 1 |
| p.K168_N192del | 503_580del | 1 |  |  |  |  | 1 |
| p.V192_I194del | 574_582del | 1 |  |  |  |  | 1 |
| p.Y186_F191del | 556_573del |  |  |  |  | 1 | 1 |
| p.Y190_V192del | 569_577del | 1 |  |  |  |  | 1 |
| **Non-synonymous substitution** |  |  |  |  |  |  |  |
| p.P216H | 647C>A |  | 4 |  | 4 | 4 | 12 |
| p.F191L | 573T>G | 1 |  |  |  |  | 1 |
| p.N193S | 578A>C | 1 |  |  |  |  | 1 |
| p.I194F | 580A>T | 1 |  |  |  |  | 1 |
| p.F191I | 556T>A |  |  | 1 |  |  | 1 |
|  |  |  |  |  |  |  |  |
| **Total number of HCA cases** |  | 43 | 256 | 25 | 241 | 361 | 926 |

1. Calderaro, J. *et al.* Molecular characterization of hepatocellular adenomas developed in patients with glycogen storage disease type I. *Journal of hepatology* **58**, 350-357 (2013).

2. Pilati, C. *et al.* Genomic profiling of hepatocellular adenomas reveals recurrent FRK-activating mutations and the mechanisms of malignant transformation. *Cancer cell* **25**, 428-441 (2014).

3. Poussin, K. *et al.* Biochemical and functional analyses of gp130 mutants unveil JAK1 as a novel therapeutic target in human inflammatory hepatocellular adenoma. *Oncoimmunology* **2**, e27090 (2013).

4. Rebouissou, S. *et al.* Frequent in-frame somatic deletions activate gp130 in inflammatory hepatocellular tumours. *Nature* **457**, 200-204 (2009).
